# In the end, it is the word that remains: communicating bad news in pediatric oncology

**DOI:** 10.1007/s00520-026-10407-5

**Published:** 2026-02-07

**Authors:** Theresia Krieger, Remo Kamm-Thonwart, Tobias Daebritz, Kerstin Dittmer

**Affiliations:** 1 Medical Psychology, Neuropsychology and Gender Studies Center for Neuropsychological Diagnostics and Intervention (CeNDI), Faculty of Medicine and University Hospital Cologne, University of Cologne, Kerpener Str. 62, 50937 Cologne, Germany; 2 Sonnenstrahl e.V. Dresden - Support Organization for Children and Adolescents with Cancer, Dresden, Germany; 3 Department of Pediatric Hematology and Oncology, Department of Pediatrics, Faculty of Medicine and University Hospital Carl Gustav Carus, Technische Universität Dresden, Dresden, Germany

**Keywords:** Breaking bad news, Communication, Health system development, Education, Pediatric oncology, Shared decision-making

## Abstract

**Purpose:**

Breaking bad news (BBN) in pediatric oncology is emotionally complex and often poorly supported by training or guidelines. The OKRA-Compass, developed through participatory research, provides practical recommendations aiming to enhance BBN quality in German pediatric oncology settings. This paper aims to track insights into the everyday BBN routine.

**Methods:**

After 6 weeks implementing the OKRA-Compass in five pediatric oncology clinics, a focus group and written feedback captured user experiences. Using thematic analysis, researchers coded and analyzed data to assess perceived changes. The participatory approach included co-researchers, and findings were linked to the Compass’s Delphi-based theses for deeper insight.

**Results:**

Applying the OKRA-Compass revealed four key outcomes: (1) Considering BBN as a complex process, (2) addressing the multi-layered needs of BBN receivers, (3) encouraging new paths for shared decision-making, and (4) cultivating awareness of the needs of healthcare professionals. Interdisciplinary use enhanced preparation, communication, and emotional safety while highlighting the need for structured training and institutional anchoring of BBN practices.

**Conclusion:**

The OKRA-Compass supports high-quality, individualized BBN by structuring communication, fostering emotional attunement, and promoting shared decision-making. It enhances interdisciplinary collaboration and self-reflection among healthcare providers. Findings highlight its practical relevance, though broader evaluation is needed. The tool offers a promising framework for improving communication culture in pediatric oncology. This process demands a readiness to adapt, allocate resources, and invest in enhancing team communication skills.

## Irreversible: the challenges of breaking bad news in pediatric oncology

In her poem *Unaufhaltsam* (Irreversible), the German lyric poet Hilde Domin posits the theory that any verbal utterance leaves an indelible mark upon the memory of those who receive it [1]. This notion becomes especially salient in the context of the physician’s communication of a cancer diagnosis to pediatric patients and their families.

Breaking bad news (BBN) refers to conversations that may change the patient’s future view in a drastic, life-changing way [2]. This may include a cancer diagnosis, disease progression or recurrence, therapy failure, or shifting treatment goals from curative to palliative [3]. Effective communication among the interdisciplinary team of healthcare professionals (HCPs), the patients, and their families (as receivers) is indispensable during BBN for exchanging information bidirectionally, building or sustaining trust and therapeutic alliance, and supporting shared decision-making [4, 5]. High-quality BBN processes have been shown to enhance receivers’ ability to process information, adjust to their diagnosis, adhere to treatments, alleviate distress, and enhance psychological well-being [5, 6].

In pediatric oncology, BBN is highly complex and requires effective communication among HCPs, patients, and their parents or other supporters. This collaborative process is often referred to as a “trialogue,” highlighting the informed and joint discussion among all parties involved [3, 7–10].

### The transmission of bad news

BBN demands high sensitivity to the unique dynamics between pediatric patients and the individuals who provide them with support [11]. This support may be provided by parents, other family members, or other individuals, who are termed “supporters” in this article. Despite its critical role in the patient care trajectory, many HCPs feel inadequately prepared for BBN [4, 7]. A multinational survey across 40 countries revealed that only 35% of healthcare professionals had undergone formal training in BBN [12]. Outpatient HCPs indicated a lack of knowledge, experience, and/or skills to facilitate the trialogue successfully and appropriately [13].

HCPs perceive BBN as considerably more stressful in pediatric oncology compared to adult oncology as a trialogue is compulsory [7, 8], which is experienced as demanding since it requires specific skills [8, 14, 15]. Canadian and Swedish studies in pediatric oncology have shown that many clinicians view BBN as challenging and often feel uncomfortable, vulnerable, or insufficiently prepared [7, 10].

Few studies comprehensively explored the needs of both HCPs and BBN receivers [14, 16]. Unsatisfactory BBN experiences can cause secondary trauma in HCPs, potentially leading to compassion fatigue, burnout, or avoidance of communication [4].

### The reception of bad news

Receiving a life-threatening diagnosis can overwhelm both children and their supporters, leading to emotional and cognitive symptoms such as anxiety, despair, attention deficits, or memory problems. It may cause withdrawal, freezing, frantic escape reactions, and disorientation—often described as a shock state [17]. This state impairs BBN receivers’ ability to process information and engage in shared decision-making. Reactions during the shock state often leave families struggling to reconcile the diagnosis with their worldview, diminishing their ability to understand medical details and engage in critical decisions [18]. Inadequate guidance, a lack of time, and insufficient BBN communication skills can potentially adversely affect pediatric patients and their families [19]. HCPs can help mitigate the effects of shock by building trust, communicating in a receiver-friendly way, and responding appropriately [20, 21].

### The requirements for breaking bad news in German pediatric oncology

In German pediatric oncology, systematic research, preparation, guidance, or tools offering high-quality BBN are lacking, and the comprehensive understanding of all parties involved in BBN is limited [3, 8]. Some international studies have addressed the receivers’ requirements [7, 22], but only one German study has explored young cancer patients’ experiences and wishes regarding BBN in clinical settings [23].

The ability to acquire trialogue communication skills is well documented; however, this topic receives insufficient attention in medical curricula and professional training for HCPs [4]. Unlike adult oncology, pediatric oncology lacks established evidence-based communication training guidelines and protocols [8, 24].

## Developing an orientation compass for BBN in pediatric oncology based on practical evidence

To support interdisciplinary teams and advance BBN practices in pediatric oncology, the research project OKRA (Orientation Compass for Breaking Bad News in Pediatric Oncology) was undertaken, funded by the German Leukemia and Research Aid (*Deutsche Leukämie und Forschungshilfe e.V.*). The project aimed to develop a comprehensive instrument (Compass) to support HCPs in the preparation, transmission, and follow-up of BBN in pediatric oncology [24].

**Fig. 1:**
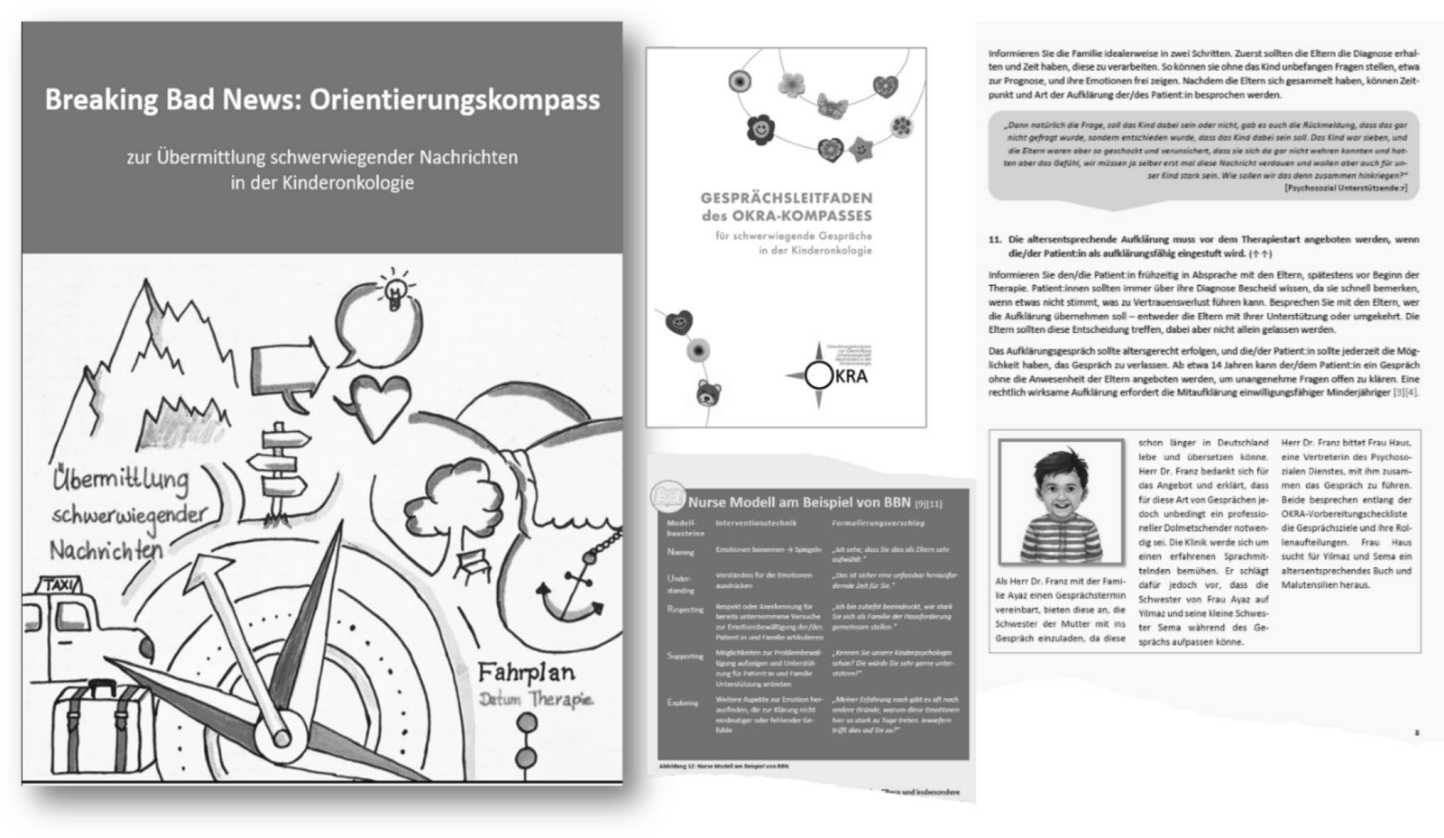
Cover of the OKRA-Compass, its pocket-sized BBN-communication guideline, and its outline

The OKRA-Compass was developed in two phases. In phase 1, high-quality BBN theses (recommendations) were formulated through a participatory group Delphi [25]. The multi-perspective, practice-based knowledge was generated by representatives from 19 organizations belonging to one of four groups: HCPs and BBN receivers, family support organizations, and researchers. In phase 2, the pilot Compass was refined and further developed in practice at five pediatric oncology clinics in Germany based on the theses [26]. The final Compass contains 122 onco-pediatric theses (recommendations), case scenarios describing the “ideal” course of the BBN conversation, illustrative quotes, information boxes, and a glossary (Fig. 1).

It can be downloaded at https://www.kinderkrebsstiftung.de/downloads/okra-kompass/.

### Purpose

This paper aims to elucidate how the availability of the OKRA-Compass has engendered novel developments or alterations in the everyday BBN routine in German pediatric oncology.

## Methods

Once the OKRA-Compass had reached maturation [26] a workshop on how to use it was offered to the project stakeholders (08/2024). Subsequently, interdisciplinary teams utilized the compass during BBN in the five clinics that participated in phase 2.

### Data collection

After 6 weeks, a focus group discussion (FGD) was conducted through a secure ZOOM channel, provided by the University of Cologne. The FGD lasted 90 min, and the main question posed to the participants was, “What was different when using the OKRA-Compass?” To engage with the participants, the chat function and storytelling were utilized. Notes were taken during the discussion. However, some participants were unable to attend the meeting due to work commitments and shared their user experiences via email.

The FGD was facilitated by the first and last authors, who are trained in nursing, public health, and participatory research. As OKRA has a participatory nature, two participants were engaged as co-researchers (DT and KTR).

### Participants

The socio-demographic data of the participants can be found in Table 1.

**Table 1:** Sociodemographic data of the participants

Sociodemographic charactaristics (*n* = 24)	Subgroup	Frequency	Percentage %
Age	30–34 years	4	17
	35–39 years	2	8
	40–44 years	2	8
	45–49 years	9	38
	50–54 years	1	4
	55–59 years	2	8
	> 59 years	4	17
Gender	Male	10	42
	Female	14	58
Federal state of Germany	Baden-Württemberg	2	8
	Bavaria	3	13
	Hesse	1	4
	North Rhine-Westphalia	11	46
	Rhineland-Palatinate	2	8
	Saxony	5	21
Context with BBN	Hospital	18	75
	Outpatient sector	3	12.5
	Self-help	3	12.5
Years of experience in the field	< 5 years	6	25
	5–9 years	4	17
	10–14 years	1	4
	15–19 years	3	13
	20–24 years	4	17
	25–29 years	2	8
	> 29 years	2	8
	Missing	2	8

### Data analysis

Two researchers (DK, KT) coded and analyzed the notes of the FGD and the written user experiences using MAXQDA software [25]. Thematic analysis, suitable for detailed information, was applied, including familiarization with the data, inductive coding, category building, topic searching, verification, topic specification, and preparation of analysis results (DK, DT, KTR, KT). The OKRA-Compass represents the consensus of the Delphi process and was refined using participatory action research. The theses of the Compass were therefore included in the analysis, and the statements from the focus group discussion were augmented.

### Ethics declaration and consent to participate

This study was approved by the Ethics Committee of the Faculty of Medical at the University of Cologne (No. 23–1187) and adhered to the principles of the Declaration of Helsinki and relevant national and European data protection regulations. Before data collection, the researchers provided written and oral information to each participant, explaining the study’s procedures and objectives. Written informed consent was then obtained from each participant.

## Results

Utilizing the OKRA-Compass in daily routines has yielded several critical insights, which can be grouped into four domains: (1) Considering BBN as a complex process, (2) addressing the multi-layered needs of BBN receivers, (3) encouraging new paths for shared decision-making, and (4) cultivating awareness of the needs of HCPs. The following sections discuss those insights.

### Considering BBN as a complex process

HCPs appreciated that the OKRA-Compass divided BBN into three phases: preparation, transmission, and follow-up. Participants observed that comprehensive preparation and follow-up led to the BBN being better perceived by both HCPs and BBN receivers. Participant 11 emphasized the importance of “assuming different perspectives and thinking about a consultation in advance.” Concurrently, HCPs recognized the significance of allocating dedicated time for structured follow-up to address the needs of both BBN receivers and HCPs: “With the compass, we allowed ourselves more time to discuss the family individually in the preparation and follow-up phases.” [P 2].

The participants perceived the methodological approach of communicating the diagnosis and medical findings in a first step and discussing the therapeutic interventions in a second step as positive in terms of preparing the conversation, communicating the content, and providing emotional support for both families and providers: “Dividing the consultation into two parts is a positive experience for the families and simplifies preparation.” [P 1].

HCPs highlighted that interdisciplinary collaboration is vital for ensuring the delivery of high-quality, personalized care to patients. This notion is also underpinned in the OKRA-Compass: “The BBN conversation must be conducted on an interprofessional basis. It is essential that at least one other profession is involved alongside the medical service (e.g., nursing, psychosocial service, clinical ethics and, if necessary, pastoral care). The selection of participants in the follow-up discussion can also be adapted to the needs of the family.” (Thesis 6).

HCPs experienced an increase in their sense of skills upon becoming cognizant of the diverse communication categories, including both verbal and non-verbal (Thesis 38). Furthermore, participants asserted that the utilization of structure aids (e.g., checklists) was pivotal in elucidating significant issues, including the need for translators, and in allocating tasks and responsibilities (Thesis 33).

### Addressing the multi-layered needs of BBN receivers

The OKRA-Compass proclaims a personalized BBN approach. Therefore, establishing a comprehensive framework encompassing the patient’s needs, resources, desires, preferences, and concerns, along with those of their family, seems crucial in fostering a more nuanced understanding of the situation. The OKRA-Compass inspires HCPs to invest courage and clarity in their communication, offering families trustworthy support and fostering hope and confidence (Thesis 30 & 44).

In BBN conversations, participants considered it crucial that patients and their families gain a general orientation about the situation. Therefore, they used methods such as Keep It Short and Simple (KISS) (Compass, p. 27) or visualizing the trajectory steps.

The HCPs reported feeling better equipped to make credible assertions, such as “there are options and expertise to deal with the cancer” [P 7], which may permit patients and their families to allay the fears of those around them and contribute to developing their coping mechanisms. HCPs mentioned that the formulation aids, which were integrated into the OKRA-Compass (p. 16) and OKRA Pocket Size Communication Guideline (Compass, Annex), prepared them to use clear and receiver-friendly language and the thoughtful use of words.

HCPs experienced recommendations regarding timing with children (Compass, p. 23) and rules for talking with children (Compass, p. 32) as helpful.

Participants considered it important to pay greater attention to welcoming emotions and responding accordingly. The HCPs found practical guidance in the OKRA-Compass on demonstrating empathy and providing effective support (Theses 36–40). Participants considered promoting moments of silence crucial to understanding the message and its consequences and addressing the emotional overload of the BBN receivers. One participant recommended that “in BBN, difficult questions should be dealt with as openly as possible” [P 10].

### Encouraging new paths for shared decision-making

During the development phase of the OKRA-Compass, HCPs prioritized including pediatric patients as participants in the BBN. After thoroughly evaluating the associated benefits and drawbacks, participants determined that this substantial issue should be addressed through a flexible recommendation. Participants agreed that the initial consultation is envisioned as a concise and mandatory meeting with the patient’s supporters, with the option for the child’s voluntary attendance and possible departure at any time.

As formulated in thesis 65, “supporters and patients should be explicitly encouraged to ask questions.”

HCPs felt encouraged to apply open questions to ascertain the family’s current level of information, identify any questions they may have, and determine their expectations of the conversation.

When starting the conversation, participants recommended the “taxi principle” (Compass, p. 30). It implies that if you want to “pick up” the family in their emotions or knowledge, you first need to know “their location.” The participants perceived the “taxi principle” as central: “It was vital for getting families on eye level so they can be involved both emotionally and in terms of content” [P 8]. HCPs experienced that it was a good base for shared decision-making processes, also recommended in Thesis 46.

### Cultivating awareness of the needs of the HCPs

Participants reported, through the availability of the OKRA-Compass, that they felt encouraged to introspect and reflect on their values (e.g., religion, culture, and beliefs) and to anticipate problem-solving strategies.

Conducting BBN as an interdisciplinary team rather than alone was experienced as meaningful, as suggested in Thesis 55, and it helped clarify and keep the roles (Thesis 54). Physicians emphasized that psychosocial services did help to clarify misunderstandings, create pauses, encourage questioning, monitor the emotional situation, reframe complex issues, welcome and manage emotions, moderate discussions, support child-appropriate communication, promote decision-making, and provide emotional support for both HCPs and BBN receivers. Similarly, nurses said that the presence of interdisciplinary team members helps to ensure first-hand professional information. A participating nurse reported a change: “Nursing is (like in old times) invited to participate in the BBN consultations on the ward” [P 6]. Involving an interdisciplinary team was experienced as helpful in ensuring BBN quality.

The participants pointed out that BBN requires certain skills. However, intensive training in this area is not regularly provided during medical school. Therefore, Thesis 41 indicates that HCPs “should be trained in conducting conversations and have communicative competencies (skills)” and that “BBN knowledge should be an integral part of further training” (Thesis 42). In addition, HCPs perceived emphasizing methods like active listening (Compass, p. 15) and the NURSE model (Compass, p. 28) as inspiring.

Developing these skills leads to improved communicative competence and assurance: “communication guideline and techniques, gives healthcare providers a sense of security.” (P 8). Even with the availability of the comprehensive compass, most HCPs in the FDG emphasized the need for comprehensive communication training as important to achieving effective communication, also mentioned in Thesis 120. One participant reported that today “the preparation is based on professional experience in BBN conversations, as well as through learning from experienced colleagues” (P 11).

Thesis 87 addresses debriefings. Thesis 110 demands that “clinics must structurally anchor and reward peer support in the difficulties of the BBN process, supervision and the follow-up of discussions.” Participants stated that a strong, unified, consistent message among all interdisciplinary team members is essential for the care trajectory. The interdisciplinary transmission approach allows for feedback among members about performance, which “may prevent secondary trauma” (P 4).

## Discussion

**Fig. 2:**
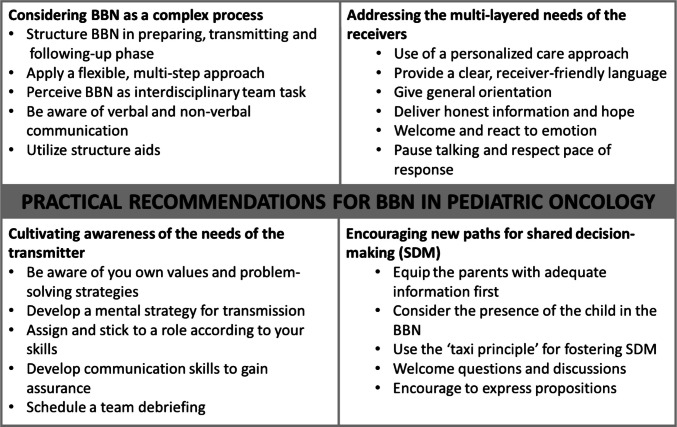
Practical recommendations for BBN in pediatric oncology

To elucidate how the availability of the OKRA-Compass has engendered novel developments or alterations in the everyday BBN routine, the FGD and the corresponding OKRA-Compass were analyzed. Based on the four domains of crucial insights of the studies’ participants, we formulated comprehensive practical recommendations for BBN for pediatric oncology (Fig. 2).

### Considering BBN as a complex process

Our findings show that HCPs valued the OKRA-Compass for its structured approach to BBN, dividing it into preparation, transmission, and follow-up. This division acknowledges that high-quality BBN goes beyond information delivery, requiring skills like responding to emotions, managing uncertainty, and decision-making [27]. Comprehensive preparation, including developing a strategy to communicate the diagnosis, acquiring information from the receivers, and managing emotions sensitively, was considered crucial for high-quality BBN, as noted by others [28].

Perceiving BBN in the pediatric context as a multi-step process is important, as it allows HCPs to better address the complex needs of the patient’s family [29]. However, it also requires openness to multiple conversations, such as separating diagnosis delivery from treatment planning, flexibility in restructuring, and resource allocation [30, 31].

Interdisciplinary collaboration, particularly with psychosocial and nursing staff, is essential for delivering personalized, high-quality care. The collaborative approach aids in clarifying profession-specific content and promotes diverse perspectives on individual patient or family circumstances [32, 33].

### Addressing the multi-layered needs of BBN receivers

Our OKRA-Compass was perceived as supporting a personalized and family-centered approach to BBN by offering concrete guidance on addressing patients’ and families’ individual needs, preferences, and concerns. In the context of a cancer diagnosis, it offers a unique opportunity to convey positive and hopeful messages [33].

The Kansas Experiment, a landmark study by Erik Wright in 1976, demonstrated the efficacy of positive suggestions and the healing effect of words in medical emergency settings [34]. Furthermore, clear communication combined with a perceived manageability of situations strengthens an individual’s “sense of coherence” [35], facilitating better coping and resilience. We promote applying age-appropriate communication strategies, including the KISS principle and visual aids like illness trajectories, for structuring the trialogue.

### Encouraging new paths for shared decision-making

The OKRA-Compass highlights the importance of involving pediatric patients in BBN while allowing flexibility based on individual circumstances. Participants supported starting with a brief, supporters-focused consultation, giving the child the option to join or leave as needed, aligning with their rights and needs in healthcare [36]. While Compass users endorsed this approach, its practical feasibility remains to be demonstrated. Involving pediatric patients and their supporters in medical decisions is more beneficial than providing selective, ready-made information [37]. Consistent with legal mandates and existing research, families generally expressed a desire to participate in decision-making and discuss treatment options [4].

To foster shared understanding and decision-making, HCPs emphasized the value of the “taxi principle,” which supports communication on equal terms and genuine participation. Encouraging families to ask questions and discuss difficult topics openly was seen as crucial for meaningful BBN conversations. Using open-ended questions, facilitating dialogue, and tailoring propositions to specific needs (e.g., school requirements) were identified as key drivers of shared decision-making, as supported by others [4].

### Cultivating awareness of the needs of the HCPs

The OKRA-Compass was found to promote self-reflection among HCPs, raising awareness of personal values and communicative blind spots—an important aspect of critical reflection skills [38]. Pediatric patients and families value HCPs who are authentic, empathic, honest, clear, courageous, and open to questions [30, 33]. As noted elsewhere, HCPs found that developing a personal mental strategy supports both information delivery and emotional self-regulation [39].

Systematic feedback and debriefings support skill development, mutual learning, and improved joint communication quality [40]. Critical reflection within the interdisciplinary team, particularly with the departmental psychologist, can help HCPs evaluate their BBN experience and competence development [41].

Interdisciplinary involvement can contribute to communication quality, consistency, and emotional stability, potentially preventing misunderstandings and crises. Nurses may clarify ambiguous words for patients or their supporters, provide emotional support, and specifically address their needs (e.g., pausing) [42].

Systematic communication training would be significant and should include BBN-related competencies into ongoing professional education, as supported by others [43]. This should include cognitive maps, active engagement, and reflection [44, 45]. In addition to learning from experienced colleagues, collegial coaching through the critical friend approach offers valuable opportunities for reflection before, during, and after BBN [46].

### Strengths and limitations

A key strength of this study is its participatory, practice-based approach. The development and evaluation of the OKRA-Compass involved diverse stakeholders, including HCPs and self-help groups, ensuring relevance across roles and disciplines in pediatric oncology. Implementation in five pediatric oncology clinics offered valuable insights into its practical use across varied institutional contexts. Integrating multiple data sources enhances the validity and credibility of findings. Thematic analysis permitted a detailed understanding of alterations to daily BBN routines influenced by the OKRA-Compass. Illustrative quotes and close alignment with the Compass’s structured theses add contextual clarity.

However, some limitations should be considered. First, although the focus group included participants from diverse professional backgrounds and regions, the small sample size may not fully reflect the range of perspectives in German pediatric oncology. Second, the findings are based on self-reported experiences after 6 weeks of Compass use, which may be too short to capture long-term effects or shifts in communication culture. Third, the qualitative design does not yield measurable outcomes on communication effectiveness or patient/family satisfaction. Future research should include longitudinal and mixed-method studies to evaluate the Compass’s impact on patient outcomes, team dynamics, and institutional practices.

Despite its limitations, this study offers valuable insights into the practical relevance and perceived benefits of the OKRA-Compass, supporting its wide adoption as a communication tool in pediatric oncology.

## Conclusion and implications for the practice

BBN quality in pediatric oncology may be overlooked when resources and training are limited. Effective BBN requires empathy, strong communication skills, and adaptation to each patient and family, making an individualized approach essential [32]. Prioritizing BBN training and integrating it into clinical education can save resources and improve critical communication skills in clinical practice. A comprehensive, easy-to-use, and accessible BBN support tool is crucial for delivering high-quality communication. The OKRA-Compass and its pocket-sized guidebook provide a clear, structured framework for both novice and experienced HCPs. Broad acceptance is expected, as it bridges the gap between the needs of BBN receivers, HCPs, and effective communication strategies. Integrating the Compass’s recommendations into daily practice requires openness to change across all levels, as well as resource allocation. A comprehensive effort will improve BBN interactions, support a needs-based trialogue, and foster a culture of collective care by giving HCPs time for reflection and processing. Ultimately, the enduring words of HCPs should instill confidence and foster a sense of feasibility for the patient and their family.

## Data Availability

No datasets were generated or analysed during the current study.
